# The impact of the invasive *Kalanchoe* × *houghtonii* on vegetated sea cliffs of the Mediterranean coasts with endemic *Limonium* species

**DOI:** 10.1093/aobpla/plag016

**Published:** 2026-04-01

**Authors:** Joan Pere Pascual-Díaz, Jordi López-Pujol, Neus Nualart, Sònia Garcia, Daniel Vitales

**Affiliations:** Institut Botànic de Barcelona, IBB (CSIC-CMCNB), Passeig del Migdia s/n, 08038 Barcelona, Catalonia, Spain; Departament de Biologia Animal, de Biologia, Vegetal i d’Ecologia, Edifici C Facultat de Biociències, Universitat Autònoma de Barcelona, 08193 Bellaterra, Catalonia, Spain; Institut Botànic de Barcelona, IBB (CSIC-CMCNB), Passeig del Migdia s/n, 08038 Barcelona, Catalonia, Spain; Escuela de Ciencias Ambientales, Universidad Espíritu Santo (UEES), Km. 2,5 vía Samborondón, Samborondón 091650, Ecuador; Institut Botànic de Barcelona, IBB (CSIC-CMCNB), Passeig del Migdia s/n, 08038 Barcelona, Catalonia, Spain; Institut Botànic de Barcelona, IBB (CSIC-CMCNB), Passeig del Migdia s/n, 08038 Barcelona, Catalonia, Spain; Institut Botànic de Barcelona, IBB (CSIC-CMCNB), Passeig del Migdia s/n, 08038 Barcelona, Catalonia, Spain

**Keywords:** invasive species, conservation, Mother of Millions, hybrid plant, niche modelling, protected areas

## Abstract

Invasive alien plant species threaten biodiversity, especially in ecologically rich regions such as the Mediterranean Basin. Coastal communities, which host many endemic taxa, are among the most affected. One such invasive taxon is *Kalanchoe* × *houghtonii*, an allegedly artificial hybrid that, despite strong invasive potential, remains largely unrecognized as a taxon of concern in Mediterranean countries. In this study, we assess its impact on the Habitat of Community Interest ‘Vegetated sea cliffs of the Mediterranean coasts with endemic *Limonium* spp.’ at two sites along the southern coast of Catalonia (NE Iberian Peninsula). We conducted fieldwork to document population size, growth stages, and spatial overlap with native species. Our results show that *K.* × *houghtonii* form dense monospecific patches that apparently compete for space with two native *Limonium* species in southern Catalonia. We also gathered 723 iNaturalist occurrences of *K.* × *houghtonii* to map its Mediterranean distribution, confirming the presence of this taxon in 107 Natura 2000 protected sites, 58 of them within this specific protected coastal habitat. Niche modelling indicates high climatic suitability of *K.* × *houghtonii* across Mediterranean Natura 2000 sites containing this particular habitat, as well as substantial overlap with Mediterranean *Limonium* spp. occurrences obtained from iNaturalist. These findings highlight the invasive potential of *K.* × *houghtonii* and support its inclusion in national catalogues of invasive species across Mediterranean countries, calling for systematic monitoring of its spread and ecological impacts.

## Teaser

Kalanchoe ×houghtonii, an allegedly artificial hybrid species, remains largely unrecognised as a taxon of invasive concern in Mediterranean countries. Our fieldwork reported dense monospecific patches in coastal communities that apparently compete for space with native Limonium species in Catalonia. We confirm its presence in conservation areas along the Mediterranean region, many within protected sea cliff habitats. Niche modelling suggests high climatic suitability and overlap with Limonium occurrences, highlighting K. ×houghtonii invasive potential and supporting its inclusion in national catalogues of invasive species.

## Introduction

Invasive alien species (IAS) are those introduced species into a natural environment where they are not native, that establish self-sustaining populations and subsequently spread (often showing rapid population growth and high demographic performance), with significant impacts on local ecosystems (Pyšek *et al.* 2020, Soto *et al.* 2024). Invasive species have become components of the floras and faunas across the world (van Kleunen *et al.* 2015) and are regarded as drivers of native species extinctions worldwide (Bellard *et al.* 2016, Roy *et al.* 2024), rising as the second cause of species gone completely extinct since 1500. In plants, invasive species have been associated with the extinction of up to 27% of species currently listed as extinct (Bellard *et al.* 2016). The importance of addressing IAS is reflected in one of the action-oriented global targets of the Kunming-Montreal Global Biodiversity Framework (target 6), which asks for reducing the rates of introduction and establishment of IAS by at least 50% by 2030 (Convention on Biological Diversity 2022).

Plant invasions are not evenly distributed across the world. The richness of alien floras depends on many factors generally related to human activities, such as trade volume, gross domestic product (GDP) per capita, population density, habitat disturbance, and climate change (Essl *et al.* 2019). Europe, particularly its Mediterranean part, is amongst the world regions with the highest invasion potential (Bellard *et al.* 2016) and invasion threat (Early *et al.* 2016). Some of the Mediterranean habitats most affected by plant invasions are coastal communities (Tordoni *et al.* 2019, Lami *et al.* 2021), with a recent study having identified coastal areas of eastern Spain, southeastern France, Italy, and northwestern Turkey among the main invasion hotspots in the northern Mediterranean Basin (Cao Pinna *et al.* 2024).

The Mediterranean coastline extends for approximately 46 000 km and is composed of about 54% rocky shores and 46% sandy coasts (Benoit and Comeau 2005). Although covering a relatively small area—due to its mainly linear nature—coastal habitats contain numerous endemic species and provide important ecosystem services including erosion control, biodiversity conservation, and recreation values (Médail and Verlaque 1997, Blondel and Médail 2009). According to the IUCN Red List (https://www.iucnredlist.org/), 99 land plant species from Mediterranean coastal habitats are classified as Vulnerable (VU), Endangered (EN), or Critically Endangered (CR). Most of these belong to the genus *Limonium*, with 43 threatened species assessed in these categories. In the European Union alone, coastal communities of Mediterranean countries are represented across 884 protected areas (PAs) included in Natura 2000 sites (https://natura2000.eea.europa.eu/). On the other hand, the often-intricate orography of coastal habitats hinders the eradication of invasive alien plant species, which can easily colonize new sites from the highly anthropized surrounding areas. In this context, assessing the occurrence of invasive plant species in protected habitats and their potential impact on threatened native species is key to implementing effective conservation measures.

One of the most recently recorded neophytes in the Mediterranean Basin is *Kalanchoe* × *houghtonii* (Crassulaceae) (Guillot Ortiz *et al.* 2014, Mesquida *et al*. 2017, Utjés *et al.* 2021). This taxon was first reported by the horticulturist A.d. Houghton in the mid-1930s in California, from an artificial cross between *Kalanchoe daigremontiana* and *K. delagoensis*, two species native to Madagascar (Houghton 1935). It has been widely studied both from morphological (Shtein *et al.* 2021) and genomic (Pascual-Díaz *et al.* 2025) perspectives. Four different morphotypes have been reported (Shtein *et al*. 2021), which differ mainly in leaf shape and length, degree of mottling, and abundance of plantlets along the leaf margins. Morphotype A corresponds to the most invasive phenotype. Both morphotypes A and B are of purported artificial origin. In contrast, morphotypes C and D are closer to the parental phenotypes and have been reported mainly in Madagascar (Shtein *et al*. 2021). Moreover, three different cytotypes (A = 4*x*, B and C = 3*x*, and D = 2*x*) have been found, being tetraploid plants—the so-called morphotype A—the most successful invaders (Pascual-Díaz *et al.* 2025). In the Mediterranean Basin, morphotype A is the predominant naturalized form, whereas morphotype B appears to be rare and has not been confirmed as naturalized (Pascual-Díaz *et al*. 2025), despite being increasingly reported globally. The invasive capacity of *K.* × *houghtonii* is enhanced by the spread of new clonal individuals through plantlets emerging from leaf margins (Shtein *et al.* 2021, Pascual-Díaz *et al.* 2025). While this reproductive trait is shared with its parental species, they exhibit lower colonization capacity as compared to the hybrid (Guerra-García *et al.* 2015, Herrera *et al.* 2018).

The presence of wild populations of *K.* × *houghtonii* was first reported in Australia (1965) and the Bahamas (1970). Since then, this hybrid has expanded worldwide and is now present on all continents except Antarctica (Herrando-Moraira *et al.* 2020). Despite its widespread distribution, *K.* × *houghtonii* is officially listed as an invasive species only in the Australian state of Queensland (Australian Queensland Government 2014) and in the US state of Florida (FLEPPC [Florida Exotic Pest Plant Council] 2017). However, although *K.* × *houghtonii* has not been recognized as an invasive species in official catalogues elsewhere, the Mediterranean Basin—particularly coastal habitats—is one of the most affected regions by its presence (Herrando-Moraira *et al.* 2020). The first confirmed wild record in the Mediterranean Basin is that of Alacant Province in eastern Spain in 1993, which was initially identified as *K. daigremontiana* (Guillot Ortiz *et al.* 2015) until recently, when we had the chance to access the herbarium sheet. Since then, this taxon is increasingly present in mostly scientific, non-official checklists of alien flora, such as those from Algeria (Sakhraoui and Thomson 2024) or Italy (Galasso *et al.* 2024). In Spain, the genus *Kalanchoe* is included in the checklist of ‘allochthonous species liable to compete with native wildlife, alter their purity, or disrupt ecological balances’ (Spanish Government 2025). However, *K.* × *houghtonii* has not yet been officially included in any legally binding list of invasive species across the Mediterranean, which hinders the implementation of control and eradication measures by public administrations.

The ability of *K.* × *houghtonii* to outcompete native species (Gómez-Bellver *et al.* 2025) has also drawn the attention of local naturalists at two coastal sites in southern Catalonia, who observed the hybrid taxon invading populations of native statices (*Limonium* spp.), species that play an important role in the sustainability of coastal and salty inland habitats (Doğan *et al*. 2020). These two sites are located within the Habitat of Community Interest (HCI) 1240, ‘vegetated sea cliffs of the Mediterranean coasts with endemic *Limonium* spp.)’—included in the Habitats Directive of the European Union (Council Directive 92/43/EEC). The vegetation of this habitat is mainly composed of *Crithmum maritimum*, accompanied by several species of *Limonium* with a generally narrow distribution. Yet, the conservation status of this habitat is considered poor in the EU, needing thus changes in management and policy (European Environmental Agency 2013). In Catalonia, the HCI 1240 covers 595.36 ha (Catalan Government 2019), accounting for 22% of this habitat in Spain, and can be subdivided into two main zones: the north zone (from Portbou to Blanes), where this habitat forms an almost continuous band along the Costa Brava, and the south zone (from Castelldefels to northern Ebro Delta), with a more discontinuous and less uniform distribution (Balaguer *et al.* 2009). Because a Catalonia-wide impact assessment was beyond the scope of the study, and because information about the conservation status from the south zone of the HCI 1240 in Catalonia is lacking, we focused our fieldwork on the two previously mentioned southern Catalan coastal sites where *K.* × *houghtonii* is established and interacts spatially with *Limonium* populations.

By combining local field assessments of spatial interaction (as evidence of potential habitat-level impacts) with a basin-wide evaluation of climatic suitability and occurrence within PAs, we provide an integrated framework to assess both potential impacts and invasion threat for the HCI 1240 across the Mediterranean Basin. Specifically, we aimed to (i) quantify population size, demographic structure, and spatial interactions of *K.* × *houghtonii* with *Limonium* taxa at two southern Catalonia sites within the HCI 1240, (ii) map the current occurrences of this hybrid in the Mediterranean region, (iii) identify its presence within Natura 2000 PAs, and also within PAs that include the HCI 1240, and (iv) model its potential geographic distribution under current climatic conditions, with particular attention to the intersection with HCI 1240 and the occurrences of *Limonium* taxa across the Mediterranean region.

## Materials and methods

### Fieldwork in Mont-roig del Camp and Tarragona (Catalonia, Spain)

**Figure 1:**
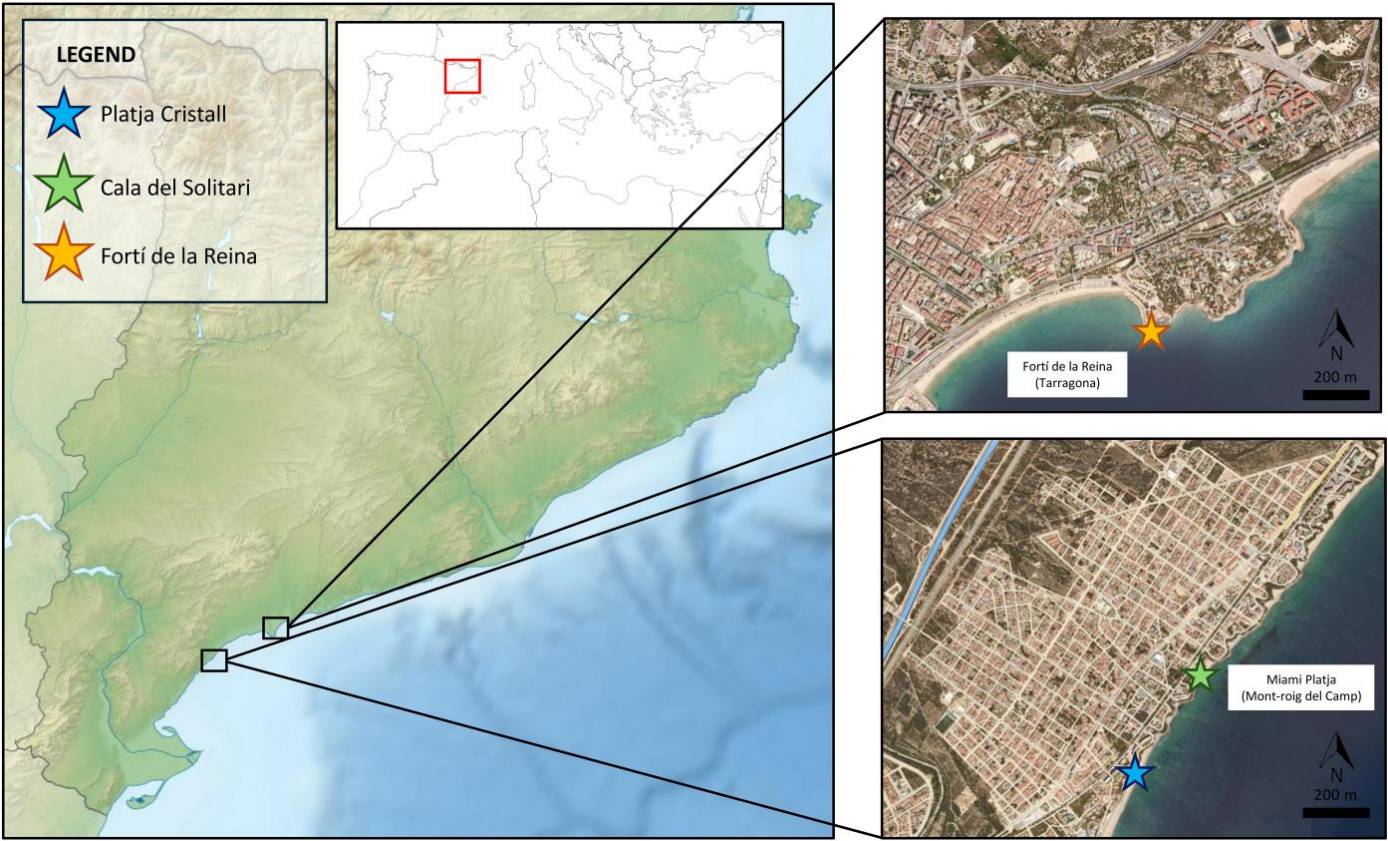
Detailed map of the localities sampled along the coast of Miami Platja (Mont-roig del Camp) and Fortí de la Reina (Tarragona). In blue, the surveyed zone on the north side of Platja Cristall; in green, Cala del Solitari; in yellow, the prospected zone of Fortí de la Reina. Credits of the maps: modified from Institut Cartogràfic i Geològic de Catalunya (Generalitat de Catalunya).

To study the level of invasion of *K.* × *houghtonii* within the HCI 1240 on the southern coast of Catalonia, we conducted fieldwork in Miami Platja (Mont-roig del Camp) and Fortí de la Reina (Tarragona) (Fig. 1), where local naturalists observed impacts of the hybrid on populations of *Limonium* spp. (Mendo *et al.* 2025). These localities contain patches of the HCI 1240, but they lie outside any Natura 2000 site or other legally autonomic or national designated protected area. Within Mont-roig del Camp, we prospected two sites: the northern side of Platja Cristall (41.00°N, 0.94°E) and Cala del Solitari (41.01°N, 0.94°E). In Tarragona, we surveyed the surrounding area of Fortí de la Reina (41.11°N, 1.27°E).

During 2022–2024, we surveyed the same coastal area every year to confirm the sustained persistence of the invasive taxon. Then, in 2025, we performed a quantitative survey (which included estimating population size, spatial extent, and demographic structure) of all *K.* × *houghtonii* patches within each locality. Fieldwork was conducted during the period of maximum vegetative activity (March–June) to facilitate species identification and the recognition of the phenological stages of *K.* × *houghtonii*. We estimated the number of individuals (as visible shoots), measured the area occupied, and recorded the distribution of phenophases. Considering the difficulty of counting individuals of *K.* × *houghtonii* because of its massive vegetative spread by propagules (implying that extremely dense monospecific patches of up to 1000–2000 individuals/m^2^ are often formed; Herrera *et al.* 2012, Tabares Mendoza 2016), the size of each population was inferred following an *ad hoc* approach. First, we counted in the field the number of cells of approximately 1 m^2^ where the species was present in the area. Then, we categorized the cells into two different types, defined based on former estimates (i.e. Herrera *et al.* 2012, Tabares Mendoza 2016) and our own *in situ* observations, as follows: (i) ‘low-density’ cells with *ca.* 250 individuals, and (ii) ‘high-density’ cells with *ca.* 1000 individuals. For each cell, we estimated the proportions of the different phenophases, defined as follows: (i) young vegetative individuals <5 cm height (considering 5 cm as the size at which they begin to produce propagules; Palerm *et al.* 2011), (ii) adult vegetative individuals >5 cm height, and (iii) individuals with inflorescences (but where plantlets are also produced, usually larger and more successful than those developed on the leaves; Tabares Mendoza 2016). Additionally, we recorded the presence of *K.* × *houghtonii* potentially competing with *Limonium* species by identifying *Limonium* taxa in the field within or adjacent to the invaded patches. Finally, for both Miami Platja and Fortí de la Reina localities, we also compiled a checklist of other invasive and potentially IAS present in the area.

### Presence of *K.* × *houghtonii* within Mediterranean protected areas and within PAs including the HCI 1240

The citizen science platform iNaturalist (https://www.inaturalist.org/) was employed to gather occurrence records along the Mediterranean Basin due to several key advantages (López-Guillén *et al.* 2024): (i) each observation carries geographical precision, (ii) all observations are accompanied by images, which help verifying correct species identification and confirming that the specimens are wild rather than cultivated, (iii) it is, by far, the largest global platform for plant observations, and (iv) occurrence data are easy to download.

A total of 1422 occurrences identified as *K.* × *houghtonii* were extracted from iNaturalist in the Mediterranean Basin (defined as lat. SW: 30.22°, long. SW: −10.87°, lat. NE: 47.69°, long. NE: 38.17°), from the first reported record to 2 February 2025. Only those categorized as Research Grade (i.e. when more than two-thirds of identifiers agree on a taxon) were considered. Then, each occurrence was manually checked to exclude those that were in cultivation or escaped but not yet naturalized (e.g. for cases such as a few individuals occurring on roofs or floor cracks). Observations with low geographic precision (>300 m) and those where the hybrid morphotype was impossible to determine were also excluded from the study. Morphotype identification was assessed based on the morphological characters (Shtein *et al.* 2021) exhibited in the pictures from iNaturalist: individuals with large, long leaves, a strong degree of mottling on the abaxial surface and abundant propagules were identified as morphotype A, whereas individuals with smaller leaves, a different mottling pattern and fewer propagules were identified as morphotype B. To ameliorate the sampling bias, the R package *spThin* was used to exclude too-close occurrences (those that clustered within 50 m, as they probably belonged to the same population). After all the adjustments, a total number of 723 filtered observations along the Mediterranean Basin were used for downstream analyses (Supplementary Table S1).

To determine the presence of *K.* × *houghtonii* in Mediterranean PAs, we first verified, using spatial data from the World Database on Protected Areas (WDPA, https://www.protectedplanet.net/), that none of the occurrences were located within a PA outside the Natura 2000 network. Then, we identified the filtered occurrences within Natura 2000 sites based on spatial data from the European Environmental Agency. Finally, to assess the susceptibility of the HCI 1240 to *K.* × *houghtonii* invasion, we had to indirectly determine it as the most recently updated HCI 1240 map (https://biodiversity.europa.eu/habitats/ANNEX1_1240) has too low spatial resolution (∼10 km) to address this question accurately. To reach this goal, we overlapped *K.* × *houghtonii* occurrences in the Mediterranean Basin with Natura 2000 sites known to include the HCI 1240; such information was obtained from the Natura 2000 Viewer (https://natura2000.eea.europa.eu/). All spatial analyses were conducted in QGIS (v. 3.36.0, Maidenhead) and R (v. 4.1.2, R Core Team 2021).

### Modelling of the distribution area of *K.* × *houghtonii* in the Mediterranean Basin

A suite of 19 bioclimatic variables was retrieved from the WorldClim 2.0 dataset (https://www.worldclim.org/) at 30 arc-second resolution, which were used for the modelling procedures. Pearson’s correlation coefficients were calculated among all bioclimatic variables to identify redundant predictors, only retaining those uncorrelated (*r* < |0.85|). For highly correlated variables, we calculated the median absolute correlation of each variable and retained those with the lowest median value. The final dataset of non-correlated bioclimatic variables included: mean diurnal range (bio2), temperature seasonality (bio4), max temperature of warmest month (bio5), mean temperature of coldest month (bio11), precipitation of wettest month (bio13), precipitation of driest month (bio14), precipitation seasonality (bio15), precipitation of warmest quarter (bio18), and precipitation of coldest quarter (bio19). In addition, a set of land-cover variables was obtained from the ESA WorldCover dataset at 0.3 arc-second resolution (WorldCover Version 2; https://esa-worldcover.org/en), including tree cover, shrubland, grassland, cropland, urban area, and bare/sparse vegetation. Land cover layers were rescaled to match the resolution of the bioclimatic variables. Afterwards, we extracted the values of the predictor variables for each occurrence point. To reduce the oversampling of the north-western Mediterranean (where the number of occurrences is much higher, probably reflecting higher iNaturalist activity and community engagement; Supplementary Fig. S1), we allowed only one occurrence per km^2^ (matching the spatial resolution of the environmental predictors, 30 arc-seconds ≈ 1 km at the equator), reaching a total number of 377 occurrences as input for the species distribution model. Two datasets of pseudoabsences with the same number of occurrences were placed randomly to calibrate the model. An ensemble of forecasts of species distribution models was then obtained, including projections from five statistical models: Generalized Linear Models (GLM), Generalized Additive Models (GAM), Multivariate Adaptive Regression Splines (MARS), Random Forests (RF), and Generalized Boosted Models (GBM). Models were calibrated using 70% randomly selected occurrence data, and accuracy was evaluated against the remaining 30% of the data, using the True Skill Statistic (TSS) (Allouche et al. 2006), and the area under the curve (Swets et al. 1988). The analysis was replicated 10 times, thus providing a 10-fold internal cross-validation of the models. To summarize all projections into a meaningful integrated projection, we employed an ensemble strategy using committee averaging based on each model’s TSS values (greater than 0.35). Models and the ensemble forecasting procedure were performed within the R package *biomod2* (Thuiller *et al.* 2009).

To better understand the potential impact of *K.* × *houghtonii* on Natura 2000 sites, as well as on the fraction of PAs being composed of the HCI 1240, we used QGIS (v. 3.36.0, Maidenhead) to intersect the ensemble projections showing over 50%, 70% and 90% suitability for the presence of *K.* × *houghtonii* with the GeoJSON file from the Natura 2000 Viewer (https://natura2000.eea.europa.eu/) containing all PAs including the HCI 1240, and by only selecting those areas within the Mediterranean bioregion. This approach will allow us to detect areas that are highly climatically suitable for invasion in the Mediterranean Basin.

To assess the spatial overlap between *K.* × *houghtonii* climatic suitability and *Limonium* occurrences across the Mediterranean Basin, we intersected the ensemble suitability projections with *Limonium* records obtained from iNaturalist in the period between 2016 and 2026 (Supplementary Fig. S5, Supplementary Table S2). Suitability maps were thresholded at 50, 70, and 90% of suitability to generate binary layers of suitable and unsuitable areas for *K.* × *houghtonii*. *Limonium* occurrences were classified as falling inside or outside suitable and unsuitable areas under each threshold, and the number and proportion of occurrences overlapping these areas were calculated by country and by Mediterranean subregion (western, eastern, and southern).

## Results and discussion

### Invasion dynamics of *K.* × *houghtonii* on the southern coast of Catalonia: Miami Platja (Mont-roig del Camp) and Fortí de la Reina (Tarragona)

**Figure 2:**
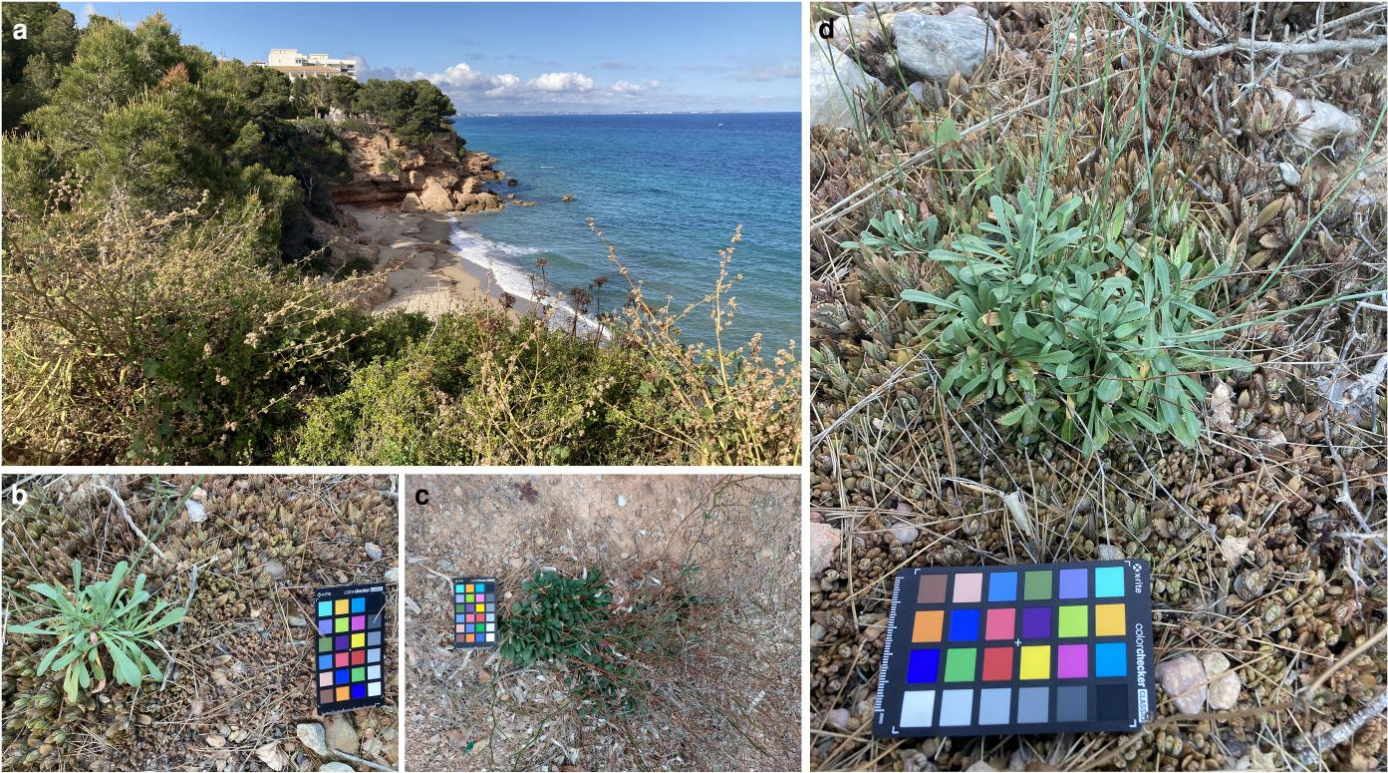
(a) Cliff ledge of Cala del Solitari (Mont-roig del Camp), where some *K.* × *houghtonii* plants with inflorescences can be observed. (b) Individuals of *L. virgatum* surrounded by a dense patch of *K.* × *houghtonii*. (c) Individuals of *L. gibertii*, a species included in the catalogue of threatened flora of Catalonia, from the site of Platja Cristall. (d) Direct competition between *L. virgatum* and *K.* × *houghtonii* in Cala del Solitari.

The results from fieldwork along the southern Catalonian coasts are summarized in Table 1. At Miami Platja (Mont-roig del Camp), specifically in Cala del Solitari (Fig. 2a), we observed the invasive hybrid covering approximately 400 m^2^, with an estimated population of 15 000 to 20 000 individuals, forming separate dense monospecific patches that partially dominate the soil cover. Most of the individuals were small plants, under 5 cm tall, but a considerable number were grown enough to start producing propagules. Larger individuals with inflorescences were in a lower proportion, being mainly restricted to the upper parts of the cliff, where they receive more sunlight, and propagules are released towards lower levels. The population structure of *K.* × *houghtonii* (Table 1), characterized by a high abundance of juvenile individuals, suggests that the population is currently expanding. Moreover, field observations reveal direct spatial co-occurrence with *L. virgatum* (Fig. 2b and d). In this context, the formation of dense juvenile carpets by *K.* × *houghtonii* in areas occupied by *Limonium* individuals supports competition for space as a plausible mechanism potentially contributing to the threat to the native species (Fig. 2b and d). However, other additional mechanisms—such as allelopathy or changes in soil properties (e.g. alterations to the soil carbon cycle), as well as shifts in the direction of ecological succession and in the composition and physiognomy of native plant communities, previously reported for *K.* × *houghtonii* in Venezuela (Herrera *et al.* 2016)—may also contribute and should be evaluated in future work.

**Table 1:** Summary of population size, area occupied, and phenological stages of *K.* × *houghtonii* in Mont-roig del Camp and Tarragona in 2025.

	Mont-roig del Camp	Tarragona
Number of individuals	15 000–20 000	130 000–140 000
Area occupied (m^2^)	400	26 650
Individuals < 5 cm	65%	60%
Individuals > 5 cm	30%	25%
Individuals with inflorescences	<5%	15%

In Cala del Solitari, populations of this hybrid invasive plant are dominated by juvenile individuals, some of which will likely develop into mature individuals that produce new propagules, potentially promoting further local expansions. This reproductive strategy results in a population structure dominated by numerous juvenile individuals, interspersed with a sizeable number of mature ones responsible of propagule production (Table 1). Given the potential further expansion of *K.* × *houghtonii*, not only could the persistence of *L. virgatum* be threatened in this site, but the conservation status of the HCI 1240 could also be negatively affected. Populations of *K.* × *houghtonii* can undergo rapid expansion following establishment, largely driven by high rates of plantlet recruitment (Herrera *et al*. 2012). This pattern has also been observed along the Costa Brava in northern Catalonia, where some populations now occupy uninterrupted stretches of coastline spanning several hundred metres, with population sizes reaching several hundred thousand individuals (Gómez-Bellver *et al.* 2025).

In Platja Cristall—the second site surveyed in Mont-roig del Camp—we detected the presence of *Limonium gibertii* (Fig. 2c), a species endemic to the Balearic Islands and the eastern coast of the Iberian Peninsula. It is listed as a protected species in Catalonia (officially classified as ‘vulnerable’; Diari Oficial de la Generalitat de Catalunya, CVE-DOGC-A-23325029-2023). Although this population is not currently threatened by *K.* × *houghtonii*, we detected a large population of the hybrid nearby (less than 100 m apart; https://www.inaturalist.org/observations/203256812). Given its ease of spread, the *L. gibertii* population in Platja Cristall could be affected by *K.* × *houghtonii* in the near future.

**Figure 3:**
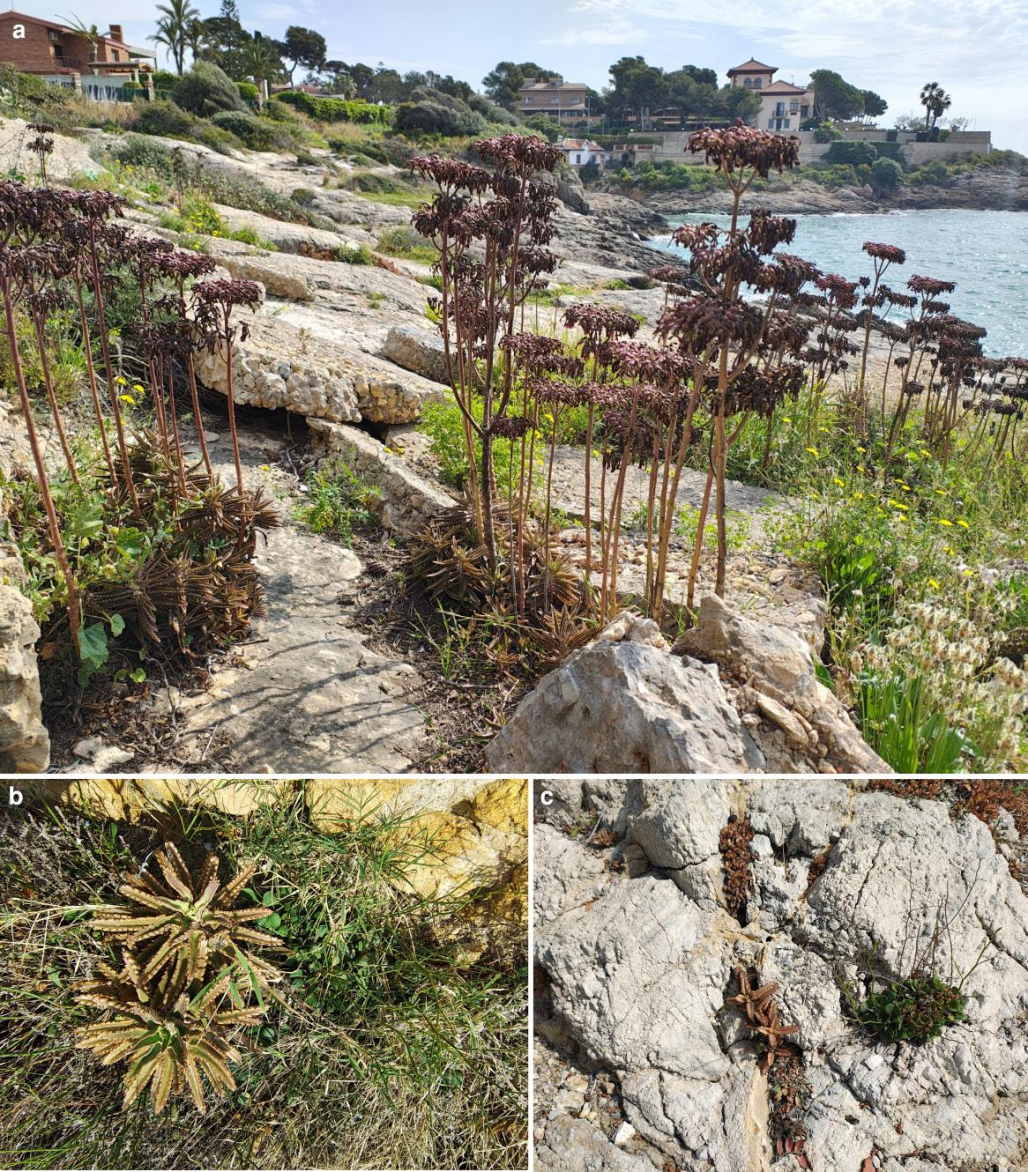
(a) Patches of individuals of *K.* × *houghtonii* with inflorescences in the coastline surrounding Fortí de la Reina (Tarragona). (b) Direct competition for the space of *K.* × *houghtonii* with *L. virgatum* and (c) with *L. gibertii*.

In Tarragona (capital), we surveyed the area surrounding the historical building of Fortí de la Reina, a bastion built in the eighteenth century. There, we found a population of *K.* × *houghtonii* roughly six times larger than the one in Mont-roig del Camp, covering an estimated area of 26 650 m^2^ and showing 130 000–140 000 individuals (Table 1). Again, the population predominantly consisted of individuals shorter than 5 cm, although a considerable number of individuals capable of producing propagules were also found. Compared to the population found in Cala del Solitari (Mont-roig del Camp), this locality had a higher proportion of individuals with inflorescences, potentially contributing to further extending the population by releasing larger and more successful plantlets (Tabares Mendoza 2016). The extension and abundance of *K.* × *houghtonii* in this locality could also be related to its long-term occurrence. This population (Fig. 3a) has been present for at least 11 years (according to historical imagery of Google Maps, see Supplementary Fig. S2; J. López-Pujol personal observation), and may have existed for around 30 years (J.R. Mendo Escoda, personal communication). In the surveyed area, and similar to what we found in Cala del Solitari with *L. virgatum*, the dense patches of *K.* × *houghtonii* are competing for the space with individuals of both *L. gibertii* and *L. virgatum* and, in many cases, the individuals of *Limonium* spp. were surrounded—and apparently displaced (some individuals interspersed with *K.* × *houghtonii* were decaying)—by vegetative individuals of the hybrid (Fig. 3b).

The invasive performance of *K.* × *houghtonii* populations surveyed on the southern coast of Catalonia is consistent with those previously reported in other coastal areas of the Mediterranean Basin. In Eivissa Island of the Balearic Archipelago (Palerm *et al.* 2011), *K.* × *houghtonii* was initially detected in small, isolated patches in Ses Salines Natural Park, specifically in La Revista (a small rural settlement) and Es Cavallet (a beach area with nearby residential zones). However, over time, the plant began to spread aggressively, affecting an area over 9000 m^2^ and threatening endemic and rare species such as *L. gibertii* and *Silene cambessedessi* (Palerm *et al.* 2011). In the Region of Murcia (Lahora *et al*. 2017), *K.* × *houghtonii* was first confirmed naturalized at the cliffs of the Águilas Castle, competing for space and affecting native endangered species such as *Fumaria munbyi*, *Filago ramosissima* (not currently listed as protected in the Region of Murcia, although its inclusion in the regional catalogue has been recommended), and *Scrophularia arguta*. In the Valencian Country, in the Cap d’Or flora micro-reserve, a *Kalanchoe* species—most likely *K.* × *houghtonii*, the only *Kalanchoe* reported in the area by iNaturalist and by our personal observations—has been largely recognized as a direct ecological threat to *Silene hifacensis* (Generalitat Valenciana 2008), a flagship species for Mediterranean plant conservation (Soler 2019) and an endangered Iberian-Balearic endemism.

These cases of invasion by *K.* × *houghtonii* in the Iberian Peninsula and Balearic Islands share key ecological features: the invaded habitats are typically coastal, rocky environments—often cliffs or disturbed outcrops—with sparse native vegetation and usually high sun exposure, where *K.* × *houghtonii* appears physiologically well adapted (Herrando-Moraira *et al.* 2020) and outcompetes native flora. Additionally, these sites are adjacent to urbanized or touristic areas, where propagule pressure is likely elevated due to gardening escape. This is further supported by the presence of many other non-native ornamental species co-occurring with *K.* × *houghtonii* in the surveyed localities of Mont-roig del Camp and Tarragona, indicating that the colonization of coastal habitats is facilitated by nearby public and private gardens acting as propagule sources. In the two studied localities, as also occurs in other areas of the HCI 1240 in the Mediterranean Basin (Misuri *et al.* 2024, Gómez-Bellver *et al.* 2025), there are very rich assemblages of alien plants (up to 21 taxa in Tarragona). Some of these alien plants co-occur spatially with native *Limonium* individuals (Supplementary Fig. S3), potentially competing for space. Besides potentially causing structural changes to communities, they also produce visual impacts that are also evident as some of the alien species are of big size (e.g. *Agave salmiana* subsp. *ferox*, *Opuntia stricta*), or occur in large quantities, forming eye-catching large carpets (e.g. *Carpobrotus* spp.). The massive entrance of alien plant species in the HCI 1240 may also produce a generalized native biodiversity loss, making these communities less resistant to further invasions (Tordoni *et al.* 2019).

### Distribution of *K.* × *houghtonii* in the Mediterranean Basin

In the Mediterranean Basin, a total of 723 naturalized occurrences have been recorded through citizen science via iNaturalist, with Spain being the country holding the largest number of occurrences of the invader *K.* × *houghtonii* (77.00%), followed by Italy (10.65%) and Portugal (4.84%). With a lower frequency, observations of the hybrid in the wild have also been detected in Algeria, Croatia, France, Greece, Jordan, Malta, Montenegro, Morocco, and Turkey. All observations of the invasive hybrid included in our analyses corresponded to morphotype A, which, according to previous studies, is the most invasive morphotype of *K.* × *houghtonii* (Shtein *et al.* 2021, Pascual-Díaz *et al.* 2025). However, the presence of the morphotype B is not discarded in the Mediterranean Basin since it was detected close to the natural reserve of the Cap de Creus (Spain) (Pascual-Díaz *et al.* 2025) and in Ostia (Italy; https://www.inaturalist.org/observations/162486856), although in these two localities the plant is not yet naturalized.

**Figure 4:**
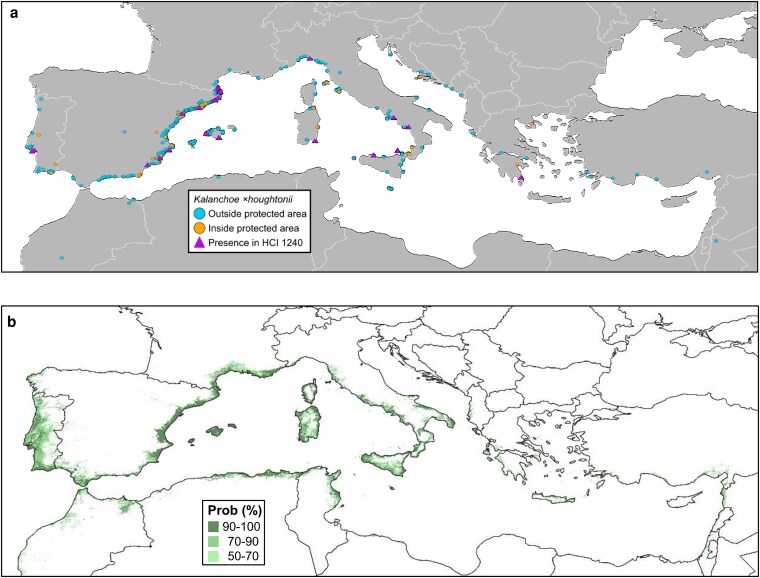
(a) Map of *K.* × *houghtonii* occurrences in the Mediterranean Basin. In blue and orange, occurrences outside and inside the Natura 2000 protected areas, respectively. In purple, occurrences inside Natura 2000 protected areas and in the habitat of community interest ‘Mediterranean cliffs with endemic *Limonium* spp.’ (HCI 1240) (b) Projections of the ensembled potential distribution models of *K.* × *houghtonii* in the Mediterranean Basin. Colours denote probabilities from 50% (light) to 100% (dark).

There is a major west–east decreasing trend in the number of wild observations of *K.* × *houghtonii* in the Mediterranean Basin (Fig. 4), a pattern that could be explained by different factors. Firstly, the earliest documented occurrence of *K.* × *houghtonii* in the western Mediterranean region dates back to 1993 (Guillot Ortiz *et al.* 2015, Pascual-Díaz *et al.* 2025), whereas the first confirmed record in the eastern Mediterranean—specifically in Greece—was much more recent, in 2016, based on a citizen science observation (https://www.inaturalist.org/observations/7405228). Therefore, assuming that the year of first observation could reflect the actual timing of arrival, the hybrid taxon may have had much more time to expand in the western than in the eastern Mediterranean. Secondly, the highest number of observations in iNaturalist from western Mediterranean countries (Supplementary Fig. S1) could reflect a more intense iNaturalist participation rather than a real higher presence of the species; in other words, the occurrence of a given species could be underestimated in countries in which people use iNaturalist much less. Thirdly, it should be noted that citizen scientists can be biased towards charismatic, easily recognizable taxa (Petersen *et al.* 2021, and references therein); thus, species that are already known to the public (e.g. through gardening or media) are more easily recognized and therefore more often reported; in Spain, *K.* × *houghtonii* is a very popular plant widely sold in gardening stores (and is also very common as a plant exchanged among friends or acquaintances) because of its supposed anti-cancer properties. Fourthly, a better conservation status of the HCI 1240 in the eastern Mediterranean compared to the western Mediterranean Basin (European Environmental Agency 2013) has been reported. Indeed, the distribution of *K.* × *houghtonii* in the Mediterranean Basin is consistent with results from Cao Pinna *et al.* (2024), which in the current climatic conditions, France, Italy, Portugal, and Spain are the most affected areas in the European Mediterranean coast by the invasion of alien naturalized flora; therefore, the presence of the hybrid would also be consistent with the conservation status of the area which colonizes. Fifth, and last, bioclimatic conditions in the eastern Mediterranean may be less favourable for the introduction and establishment of *K.* × *houghtonii* than in the western Mediterranean (discussed further in the next section).

Most of the factors that explain the west–east gradient in the number of *K.* × *houghtonii* occurrences also account for the marked differences between the northern and southern parts of the basin: the plant arrived much later in the region (the first confirmed record dates to 2008 in Tunisia; Herrando-Moraira *et al.* 2020), iNaturalist is far less widely used, the species is little known to the general public, and the bioclimatically suitable area is very limited—restricted mainly to the coastal zones of Morocco, eastern Algeria, and northern Tunisia, while its presence in Libya and Egypt appears highly unlikely (see below). An additional factor is the comparatively lower GDP of the southern basin countries, as economic development is a widely recognized key driver of biological invasions (Hulme 2009, Pyšek *et al.* 2010).

From all observations of *K.* × *houghtonii* in the Mediterranean, 107 (14.80%) have been reported inside PAs, and these exclusively occur in the northern Mediterranean Basin (Fig. 4a), but none in North Africa. More than half of the occurrences inside PAs are also found within areas containing the HCI 1240 (i.e. a total number of 58 occurrences representing 8.02% of the whole dataset; Fig. 4a). This strongly suggests that this habitat may be particularly sensitive to invasion by *K.* × *houghtonii*. Rocky habitats are among the commonest in which the hybrid occurs around the world (Herrando-Moraira *et al.* 2020), likely mirroring those of the parental species in Madagascar, where they grow on granite, sandstone or limestone outcrops, or coastal and inland unconsolidated sands (Boiteau and Allorge-Boiteau 1995, Descoings 2003, Witt and Rajaonarison 2004), often in direct sunlight (Hannan-Jones and Playford 2002) and having adaptations to high irradiation (Kluge and Brulfert 2000). These conditions (rocky substrates and full sun exposition) are generally fully met in coastal cliffs. In addition, *K.* × *houghtonii* significantly increases plantlet production under experimental high-light conditions (Zhang *et al.* 2026), which could enhance its colonization capabilities.

Most of these observations of *K.* × *houghtonii* dwelling in the HCI 1240 inside PAs are concentrated in the western Mediterranean, with just a single observation in the eastern part of the basin (Greece) (Fig. 4a). This geographic pattern matches the conservation status of the HCI 1240 in the Mediterranean Basin, being unfavourable in its western part (Spain, France, and Italy), but favourable in its eastern one (Croatia and Greece; https://nature-art17.eionet.europa.eu/article17/habitat/summary/?period=5&subject=1240). Thus, while this pattern is compatible with lower biotic resistance (Rejmnek 1996) and a poorer conservation status of the HCI 1240 in the western Mediterranean, also likely reflects a west–east bias in biodiversity research effort in the Mediterranean Basin (García-Roselló *et al.* 2023).

### Projection of the potential habitat of *K.* × *houghtonii* in the Mediterranean Basin

To investigate the potential for future spread and identify vulnerable areas—especially within or near PAs—we conducted species distribution modelling to estimate the climatically suitable range of *K.* × *houghtonii* across the Mediterranean Basin. The projection of its potential distribution indicates that the western Mediterranean—particularly Spain and Italy—is highly suitable for the nothospecies under current climatic conditions, especially in coastal and peri-urban areas. Although recorded occurrences are currently much lower in the eastern Mediterranean (Fig. 4a), ensemble species distribution models suggest that many coastal areas in this region also present favourable climatic conditions for its establishment (Fig. 4b). Given the still low number of occurrences and the climatic suitability of the hybrid, we consider coastal areas of the eastern Mediterranean Basin as a ‘red alert’ zone—i.e. an area not yet widely colonized but highly vulnerable to future invasions (Herrando-Moraira *et al*. 2020).

Across the European Mediterranean bioregion, there are 433 Natura 2000 PAs that include the HCI 1240. Of these, 73.21% are located in the western Mediterranean, while 26.79% are in the eastern Mediterranean. According to our distribution model (at a suitability threshold of 50%), 96.15% of Natura 2000 PAs containing the HCI 1240 in the western Mediterranean are suitable for colonization by *K.* × *houghtonii* (Supplementary Fig. S4). In the eastern Mediterranean, 59.46% of such areas are suitable. This lower percentage in the east is partially due to the Natura 2000 PAs of Slovenia—four of which harbour the HCI 1240, none of them being inferred as climatically suitable for the hybrid. In contrast, Croatia, Greece, and Cyprus show suitability rates of 71.88%, 94.52% and 71.43%, respectively, across all their Natura 2000 PAs containing the HCI 1240.

When the suitability threshold is increased to 70%, the potential presence of *K.* × *houghtonii* in Natura 2000 PAs in the western Mediterranean remains unchanged (96.03%), while it slightly decreases in the eastern Mediterranean (40.77%). Finally, at a stricter suitability threshold of 90%, the western Mediterranean remains highly suitable for *K.* × *houghtonii* (94.14%), whereas suitability in the eastern Mediterranean decreases substantially (26.39%). This reduction is mainly explained by the absence of suitable climatic conditions in the PAs harbouring the HCI 1240 in Cyprus and Slovenia, as well as by a decrease of climatically suitable Natura 2000 PAs in Croatia and Greece (56.25% and 49.32%, respectively).

**Figure 5:**
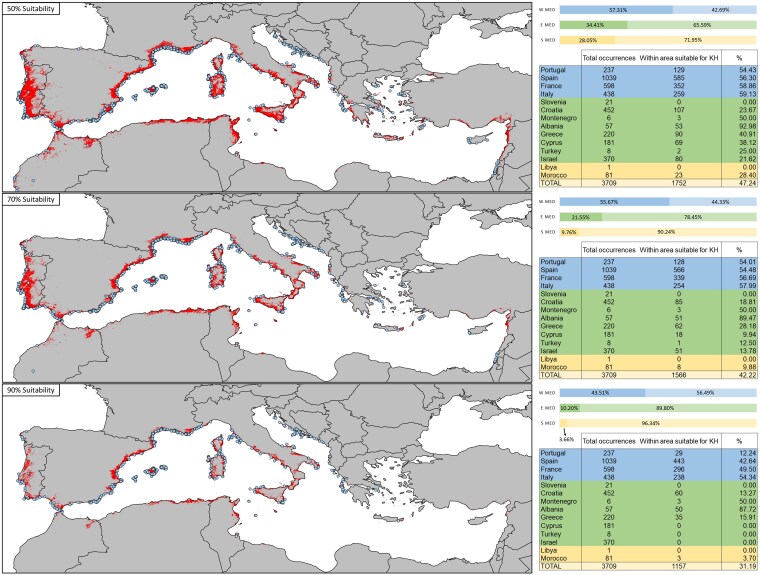
Climatic and vegetation suitability of *K.* × *houghtonii* (KH) across the Mediterranean Basin and overlap with *Limonium* occurrences from iNaturalist. Maps show areas predicted as suitable for KH under the three suitability thresholds (50%, 70%, and 90%; indicated in red). Blue dots represent *Limonium* occurrences that fall within suitable areas under each threshold. For each threshold, tables summarize the total number of *Limonium* occurrences per country and the number and percentage of those occurrences located within KH-suitable areas. Bar plots illustrate, for each threshold, the proportion of *Limonium* occurrences falling inside (darker colour) and outside (lighter colour) KH suitable areas, divided by each Mediterranean subregion (W MED = western, E MED = eastern and S MED = southern).

When considering *Limonium* occurrences (Fig. 5), at the 50% suitability threshold, nearly half of all *Limonium* records (47.2%) fall within areas predicted as suitable for *K.* × *houghtonii*, with the overlap decreasing to 42.2% and 31.2% under the 70% and 90% thresholds, respectively. The predicted overlap is consistently higher in the western Mediterranean (with 43.51% of *Limonium* occurrences spatially overlapping with suitable areas for *K.* × *houghtonii* at the strictest threshold) and lower in the eastern and southern Mediterranean regions (with 10.20% and 3.66% of *Limonium* occurrences at 90% threshold, respectively), reflecting the unequal distribution of suitable conditions for *K.* × *houghtonii* across the basin. These results indicate that a substantial fraction of *Limonium* occurrences appear in areas potentially suitable for *K.* × *houghtonii*.

Our results highlight the need to develop region-specific strategies to manage *K.* × *houghtonii* across the Mediterranean. In the western Mediterranean, where this invasive species is already widespread and affecting native endangered flora, management should focus on mitigating the expansion of *K.* × *houghtonii* and locally eradicating it within natural PA. In the eastern Mediterranean, where the hybrid taxon is currently less established according to available occurrence data, prevention efforts are essential to limit its spread. Our potential distribution models indicate that even under very strict suitability thresholds, half of the PAs harbouring the HCI 1240 in Greece and Croatia (countries that manage more than 90% of the Natura 2000 sites containing the HCI 1240 in the eastern Mediterranean) could still be affected by *K.* × *houghtonii*. Meanwhile, on the south-western part of the Mediterranean Basin, scarcity of records associated with limited botanical documentation and reduced citizen science activity, combined with the high habitat suitability in coastal areas (Fig. 4b), indicates that this area should be continuously monitored.

## Conclusions

Despite growing evidence of the current and potential impact of *K.* × *houghtonii* on Mediterranean coastal habitats, this taxon is not yet included in the invasive species regulations of any country in this region. In Spain, the most recent catalogue on invasive species (Royal Decrees 630/2013 and 216/2019, Orders TED/1126/2020 and TED/339/2023) includes a total number of 199 invasive taxa (114 animals, 69 plants, 15 algae, and one fungus). However, *K.* × *houghtonii* and their parental species, also known to exhibit invasive behaviour, are not included in this catalogue. Considering the ecological impacts on native species and habitats reported here and in previous studies (e.g. Palerm *et al.* 2011, Lahora *et al*. 2017, Gómez-Bellver *et al.* 2025), *K.* × *houghtonii* meets the criteria for classification as an invasive species under Spanish legislation. Its formal inclusion in the Spanish Catalogue of Invasive Species would facilitate coordinated monitoring, control, support the implementation of eradication measures, and help mitigate further ecological degradation in vulnerable habitats. In other western Mediterranean countries such as Portugal, France or Italy, although no data on the impact of *K.* × *houghtonii* on the local flora are yet available, the hybrid’s widespread occurrence and large potential distribution underscore the need to consider similar legal and management strategies. The authors of this manuscript are already working on the inclusion of *K.* × *houghtonii* in the Spanish Catalogue of IAS, a step that could facilitate the incorporation of this hybrid taxon in the legislation of other Mediterranean countries.

*Kalanchoe* × *houghtonii* is mainly propagated by vegetative dispersal through clonal plantlets, and our recommended control and eradication method for this plant is its manual removal in invaded areas (using plastic bags during removal to avoid the plantlets detaching from leaves and spreading around), followed by a reintroduction of native species if needed. This method was successfully used to recover the habitat of Gopher tortoises (*Gopherus polyphemus*) in Nassau County (Florida, US), where *K.* × *houghtonii* had invaded dune ecosystems (Hérbert 2025). Manual removal of *K.* × *houghtonii* is also being applied in HCI 1240 localities within the Parc Natural de Cap de Creus (Catalonia) (J. Bech, personal communication). Moreover, due to the current extended usage of this hybrid taxon (as well as other *Kalanchoe* species) as an ornamental plant in Mediterranean countries, we recommend banning its trade. Such a measure, linked to the inclusion of the taxon in national invasive species legislation, would help prevent and mitigate its expansion into coastal habitats.

The findings of this study also suggest that *K.* × *houghtonii* should be considered for inclusion in checklists of invasive species across Eastern Mediterranean Basin countries. In Greece, the most recent list of alien plants is the *Atlas of Alien Plants of Greece* (http://www.alienplants.gr), which currently lists 456 alien species but does not include any *Kalanchoe* taxa. A similar situation is observed in Turkey, where only two *Kalanchoe* taxa (*K. delagoensis* and *K. blossfeldiana*) are included among the country’s 340 alien plants (Uludağ *et al.* 2017). In Croatia, the national list of alien species (https://invazivnevrste.haop.hr/) includes 3171 species, among them *K.* × *houghtonii* and other species of the genus. However, the hybrid is not listed in the national blacklist of invasive species (OG 15/2018, 14/2019 from the Croatian Government), so its cultivation, commercialization, or introduction into nature is not regulated. Based on current evidence—showing that this hybrid taxon is already naturalized in protected natural areas of these countries—we advocate for systematic monitoring of the spread and ecological impact of *K.* × *houghtonii* across the eastern Mediterranean area.

## Supplementary Material

plag016_Supplementary_Data

## Data Availability

The raw data are available as supporting information.
