# Supplementary material for: The impact of the invasive Kalanchoe × houghtonii on vegetated sea cliffs of the Mediterranean coasts with endemic Limonium species
Source: AoB Plants. 2026 Apr 1;18(2):plag016. doi: 10.1093/aobpla/plag016 (PMC13056715; doi:10.1093/aobpla/plag016)
Supplement: plag016_Supplementary_Data [file plag016_supplementary_data.zip › Supporting_Figures.docx]

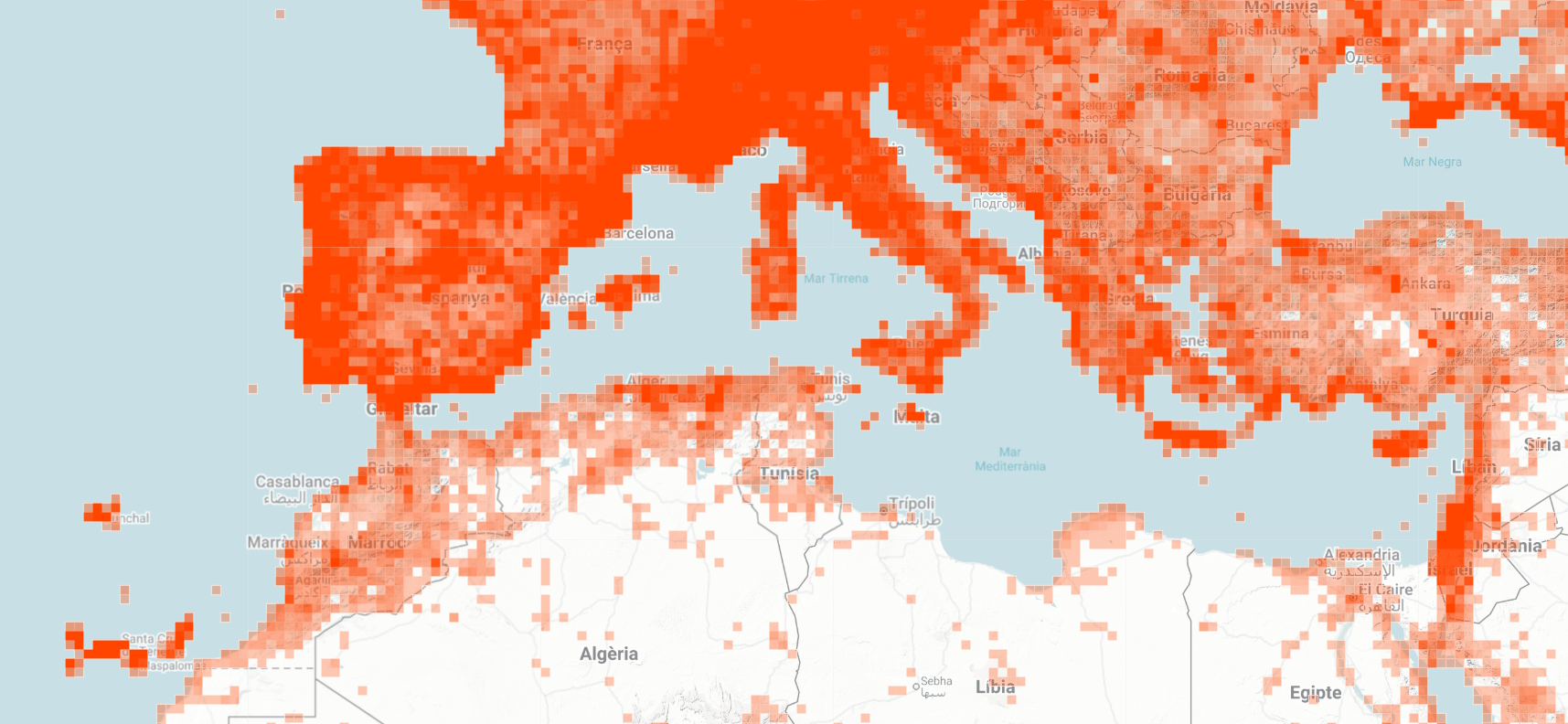
**FIGURE S1.** Screenshot (August 5, 2025) of the density of iNaturalist all plant observations (i.e., using the category ‘Plantae’ for search filter) in the Mediterranean Basin (source: <https://www.inaturalist.org/observations?subview=map&taxon_id=47126>). As iNaturalist does not offer public information on the geographic location of observers, we can take countries with the most observations as a good proxy for active participation as observers in this platform.


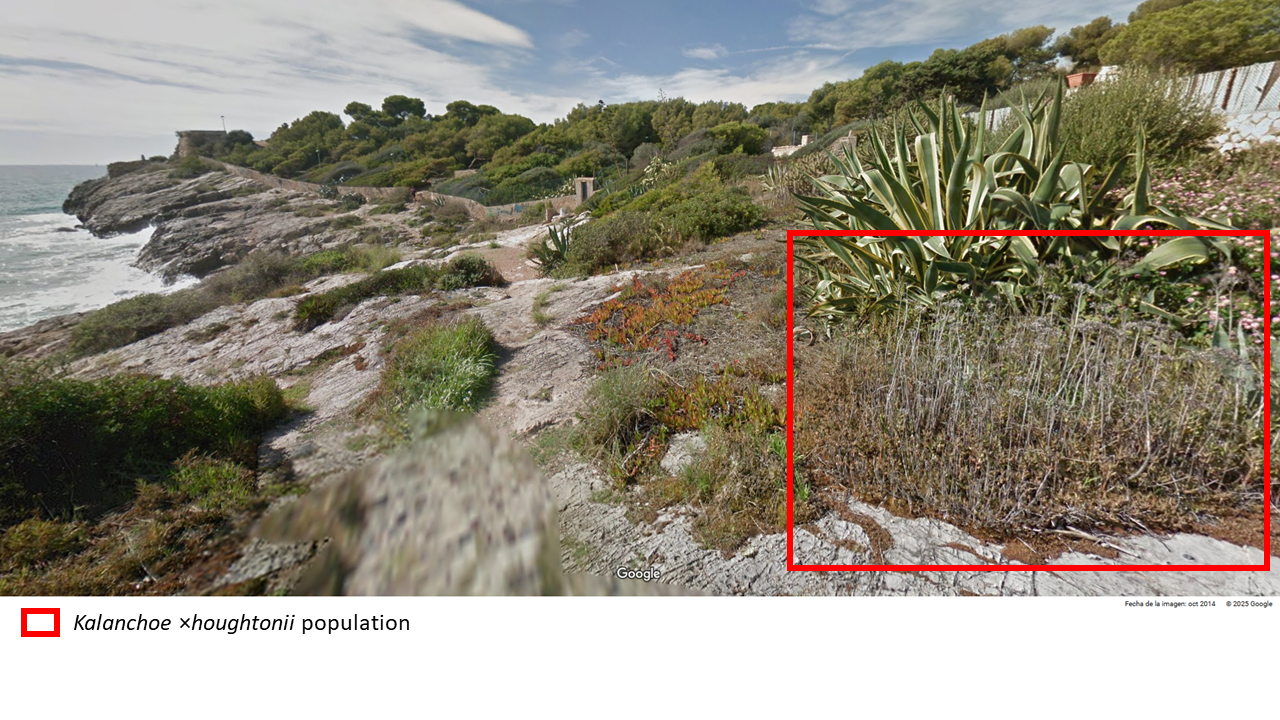
**FIGURE S2.** Picture extracted from Google Street View from October 2014 in the Fortí de la Reina (Tarragona, Catalonia, Spain) locality, with a population of *Kalanchoe ×houghtonii* established in the area.

**
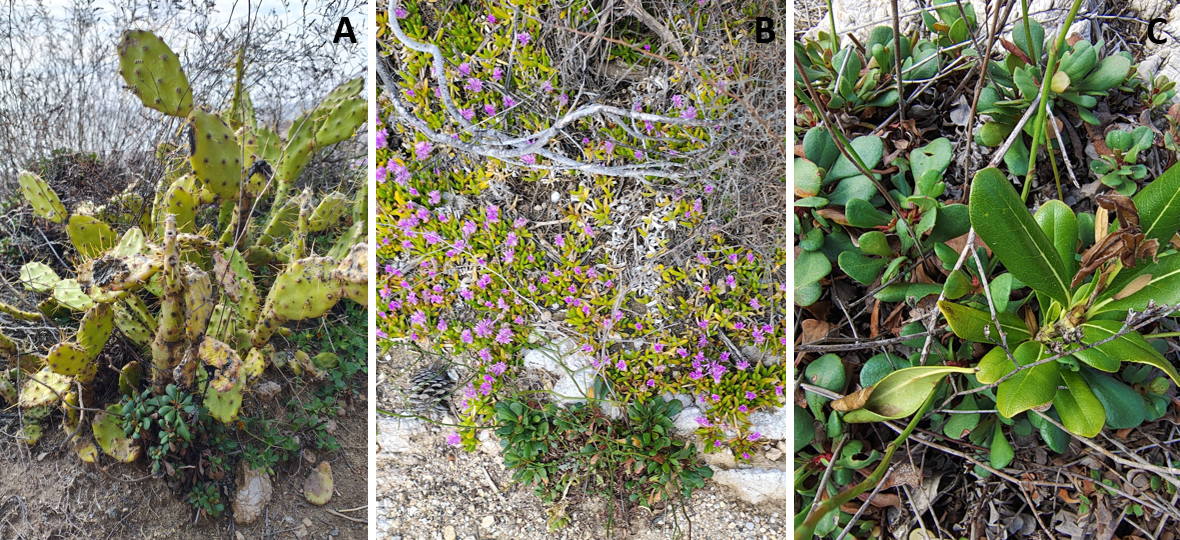
FIGURE S3.** Pictures of (A) *Opuntia stricta*, (B) *Disphyma crassifolium*, and (C) *Pittosporum tobira* competing for space with *Limonium virgatum* (indicated with a white arrow) populations in Fortí de la Reina.


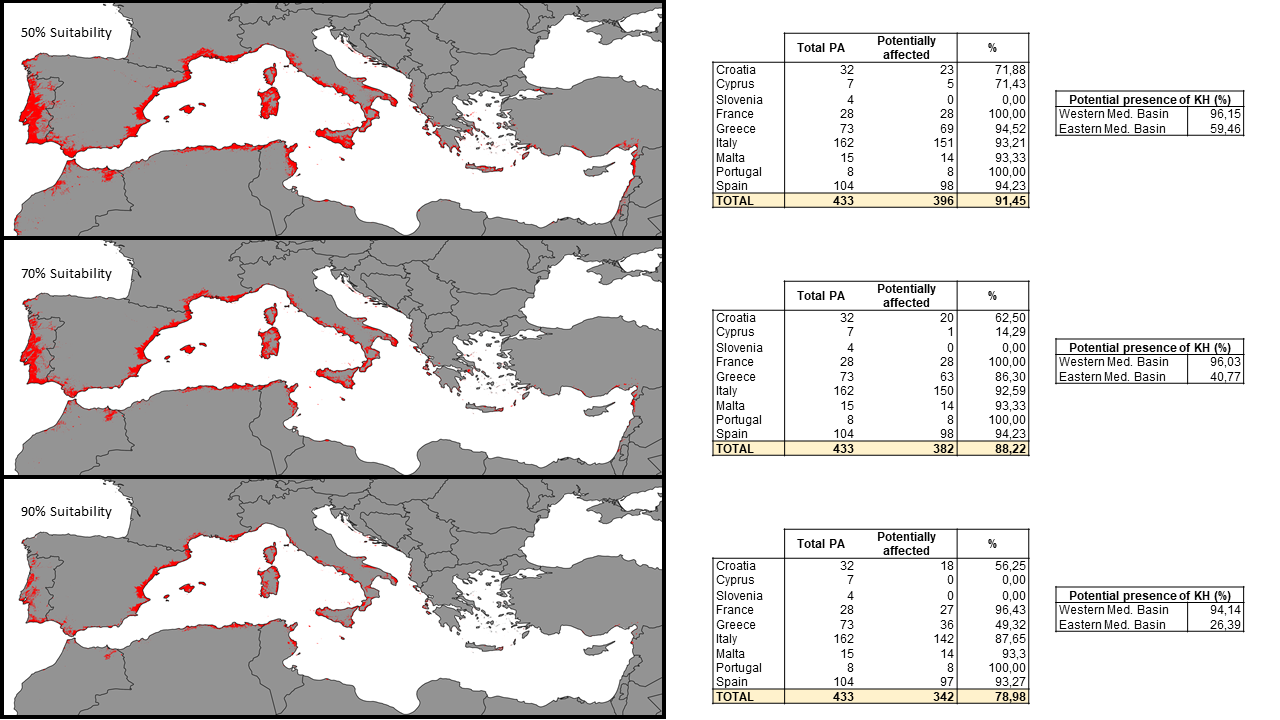


**FIGURE S4.** Predicted climatic and vegetation suitability of *Kalanchoe ×houghtonii* across the Mediterranean Basin under three suitability thresholds (50%, 70%, and 90%). Red areas indicate areas classified as suitable. For each scenario, tables report the total number of protected areas (PA) within Natura 2000 sites per country and the number of PAs potentially affected by the presence of *K. ×houghtonii*. In addition, the percentage of potential presence of *K. ×houghtonii* for each western and eastern Mediterranean Basin is indicated.


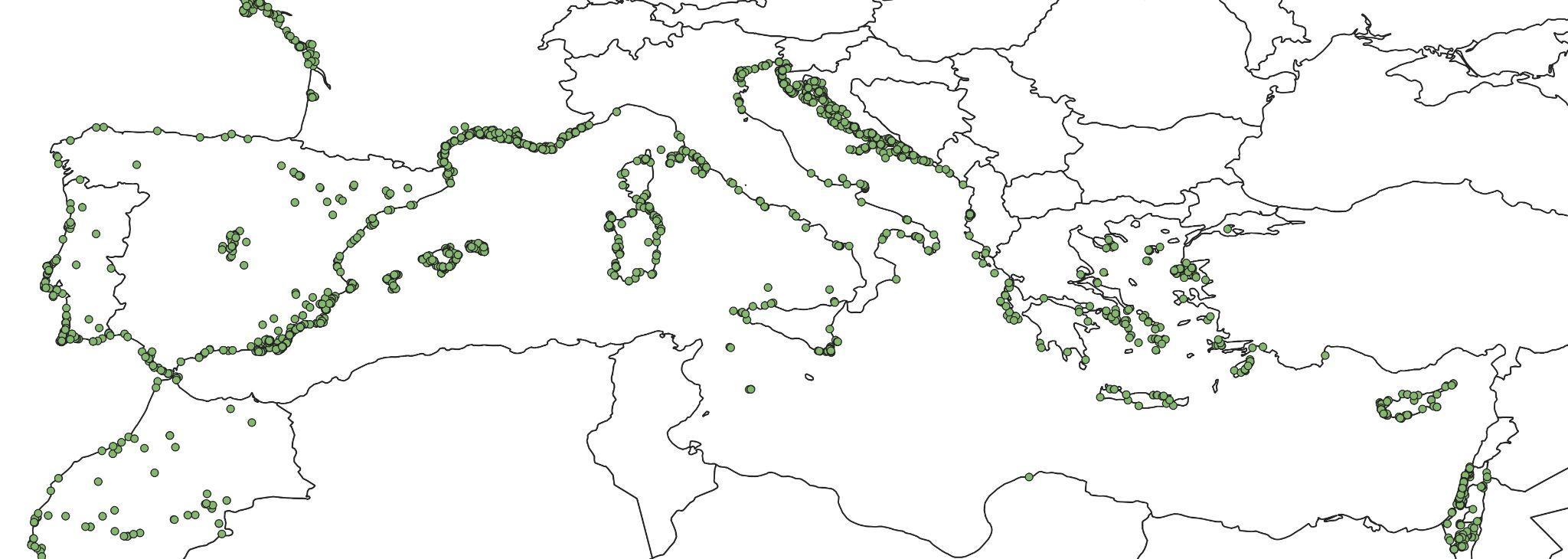


**FIGURE S5.** Occurrences of *Limonium* records on iNaturalist between 2016 and 2026, showing the spatial distribution of observations across the Mediterranean Basin.
